# Resilience in symptom response as an adaptive capacity: methodological and theoretical clarifications

**DOI:** 10.1093/oncolo/oyag410

**Published:** 2026-10-13

**Authors:** Zhihong Zhang, Marie Flannery

**Affiliations:** School of Nursing, Columbia University, New York, NY 10032, United States; School of Nursing, University of Rochester, Rochester, NY 14620, United States

We thank Geeraerts and Pivodic for their thoughtful comments.^1^ They raise three methodological concerns and question whether resilience in symptom response extends beyond a descriptive label. We welcome the opportunity to clarify our methods and theoretical contribution.

Their first concern is that the six-month observation period may have influenced the resilience classifications. This window reflected available follow-up and captured early and subsequent symptom responses, including potential recovery. As acknowledged in our limitations, different observation periods or assessment schedules may yield different classifications. Resilience in symptom response should therefore be interpreted within the specified treatment context and observation period, not as a time-invariant patient attribute.^2^^,^^3^

Second, they question our use of a composite symptom measure. We selected it to represent an individual’s overall capacity to respond to cancer treatment, as a single symptom may be insufficient when patients experience multiple concurrent symptoms. This approach is consistent with research emphasizing multiple indicators to better capture overall resilience^4–6^; for example, a National Institute on Aging workshop prioritized measures spanning multiple physiological systems.^4^ We therefore included 24 symptoms across 10 body systems. We acknowledge that aggregation may obscure symptom-specific changes and plan to compare classifications based on individual symptoms with those based on the composite score in a separate study.

Third, they question our designation of the individual as the “resilience system” and our limited consideration of contextual influences. The National Institutes of Health resilience conceptual model allows resilience to be examined at multiple levels, such as molecular, physiological, psychological, individual, community, and environmental levels.^7^ Consistent with our focus on patients’ symptom responses, we examined the individual level. To better capture factors influencing resilience at this level, we adapted the NIH model by integrating it with Kumpfer’s framework.^2^^,^^7^^,^^8^ The resulting framework identifies four categories of factors that may shape resilience: the stressor, the individual, the environment, and interactions between the individual and environment. Because its development was beyond the original article’s scope, we reported it in a separate manuscript under review and present it here for clarification (Figure 1).

**Figure 1.**
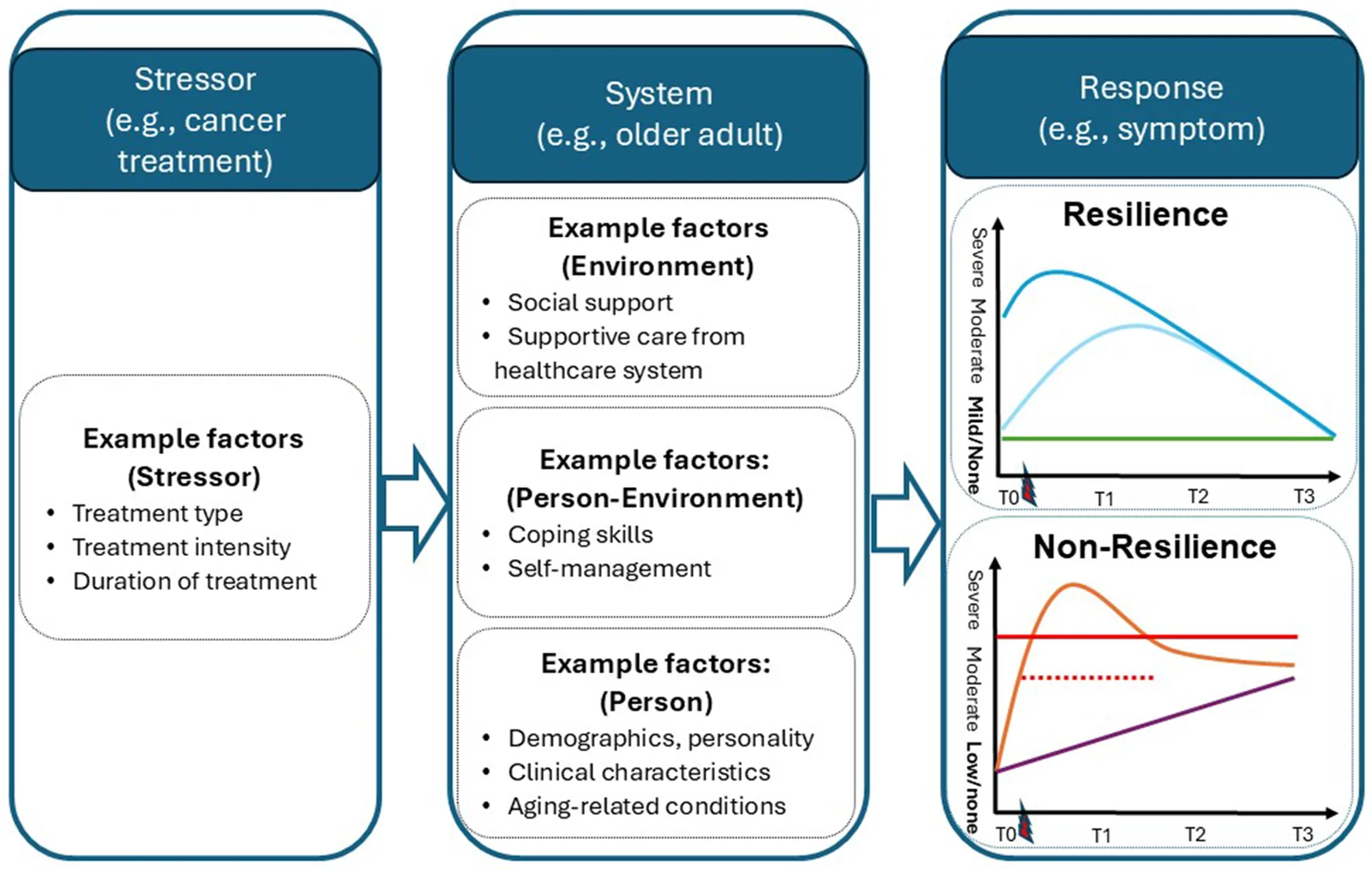
Proposed framework of resilience in symptom response. The framework integrates the stressor-system-response structure of the NIH Resilience Concept Model with individual, environmental, and person-environment interaction factors from Kumpfer’s resilience framework. In our study, cancer treatment is the stressor, the patient is the system, and symptom trajectories are the responses. Resilience is identified by trajectories demonstrating resistance, recovery, or growth/improvement within a defined observation period. Details of these trajectories are provided in the original article.^1^

Beyond these methodological concerns, Geeraerts and Pivodic raise a central theoretical concern: that we used resilience as a “descriptive label rather than an explanatory construct.” Yet identifying resilience from response trajectories is consistent with the NIH Resilience Concept Model and established methods for measuring resilience. The model defines resilience as a system’s capacity to resist, recover, adapt, or grow after a challenge; because this capacity cannot be directly observed, it is inferred from positive adaptation following a defined stressor.^7^ Thus, description is the method used to identify resilience, not the limit of its theoretical role. Its theoretical value lies in interpreting differences in symptom burden and recovery as potential manifestations of patients’ underlying capacity to respond to cancer treatment. This perspective complements existing frameworks that explain symptom experiences, influencing factors, and consequences. Our study provides proof of concept; if validated, this conceptualization may help identify mechanisms and modifiable factors and guide interventions to strengthen this capacity and improve overall symptom response.
